# Gene expression-based outcome prediction in advanced stage classical Hodgkin lymphoma treated with BEACOPP

**DOI:** 10.1038/s41375-021-01314-1

**Published:** 2021-06-10

**Authors:** Ron D. Jachimowicz, Wolfram Klapper, Gunther Glehr, Horst Müller, Heinz Haverkamp, Christoph Thorns, Martin Leo Hansmann, Peter Möller, Harald Stein, Thorsten Rehberg, Bastian von Tresckow, H. C. Reinhardt, Peter Borchmann, Fong Chun Chan, Rainer Spang, David W. Scott, Andreas Engert, Christian Steidl, Michael Altenbuchinger, Andreas Rosenwald

**Affiliations:** 1 Department I of Internal Medicine; German Hodgkin Study Group, University of Cologne, Faculty of Medicine and University Hospital Cologne, Cologne, Germany; 2 Max Planck Research Group Mechanisms of DNA Repair, Max Planck Institute for Biology of Ageing, Cologne, Germany; 3 Cologne Excellence Cluster on Cellular Stress Response in Aging-Associated Diseases, University of Cologne, Cologne, Germany; 4 Center for Molecular Medicine Cologne, University of Cologne, Cologne, Germany; 5 Department of Pathology, Hematopathology Section, University Hospital Schleswig-Holstein, Christian-Albrechts-University, Kiel, Germany; 6 Statistical Bioinformatics, Institute of Functional Genomics, University of Regensburg, Regensburg, Germany; 7 Department of Pathology, Marien-Hospital Hamburg, Hamburg, Germany; 8 Department of Pathology, University Hospital Frankfurt, Frankfurt, Germany; 9 Department of Pathology, University Hospital Ulm, Ulm, Germany; 10 Pathodiagnostik, Berlin, Germany; 11 Department of Hematology and Stem Cell Transplantation, University Hospital Essen, University Duisburg-Essen, German Cancer Consortium (DKTK partner site Essen), Essen, Germany; 12 British Columbia Cancer Agency, Vancouver, BC Canada; 13 Research Group Computational Biology, University of Hohenheim, Stuttgart, Germany; 14 Institute of Pathology, University of Würzburg, and Comprehensive Cancer Center Mainfranken, Würzburg, Germany

**Keywords:** Hodgkin lymphoma, Cancer microenvironment

## To the Editor:

Classical Hodgkin Lymphoma (cHL) is a B cell-derived lymphoid malignancy, affecting 2.5–3/100,000 people per year. To date, in patients diagnosed with advanced cHL no reliable tool is able to—a priori—distinguish the subset of patients at high risk for relapse or refractory disease. Clinical risk indices for cHL, such as the International Prognostic Score (IPS), have not been successfully applied as a treatment decision tool in advanced stage cHL [1]. In this study we show, that a previously published gene expression-based predictor in advanced stage cHL patients treated with ABVD [2] does not prove prognostic in 401 BEACOPP-treated advanced stage cHL patients. Using transcriptome profiling, we identified however that three individual genes, *PDGFRA*, *TNFRSF8* (encoding CD30) and *CCL17* (encoding TARC), were significantly associated with progression-free survival (PFS) after multiple test correction in the BEACOPP-treated cohort, highlighting the potential of a modified gene expression profiling approach for pre-treatment risk assessment.

In search of a predictive tool in advanced stage cHL patients, we have applied the previously published 23-gene gene expression‐based predictor [2], that is prognostic for overall survival (OS) in patients with locally extensive and advanced stage disease treated with ABVD chemotherapy in a distinct cohort of patients with advanced-stage cHL patients treated with BEACOPP-based regimens. In particular, we explored the relationship of the predictor score with clinical parameters and OS and PFS in the BEACOPP-based study cohort. For that purpose, we extracted RNA from formalin‐fixed paraffin‐embedded (FFPE) diagnostic biopsies of 404 patients with advanced stage cHL treated within the HD12 and HD15 trials of the German Hodgkin Study Group [3, 4]. Three samples were flagged as low quality and removed from subsequent analysis. Inclusion criteria were the documentation of a reference histology result, the availability of an FFPE lymph-node specimen obtained at first diagnosis, and a complete documentation of present prognostic factors [3, 4]. To increase statistical power for observations linked to treatment outcome, the patient cohort was enriched for progression, relapse or death events at a ratio of 1:2. Patient characteristics of the 401 patients are displayed in Table 1A. A majority of this cohort has been previously utilized for an a priori risk assessment by whole-slide image analysis [5].

**Table 1:** Patient characteristics of 401 advanced stage cHL patients treated within clinical trials HD12 and HD15 and prediction analysis of the adjusted gene predictor.

A. Patient characteristics with risk groups resulting from the adjusted gene predictor						
	All		High risk		Low risk	
	*N* = 401		*N* = 149		*N* = 252	
	*N*	%	*N*	%	*N*	%
Age (years)						
Median	33		39		29	
Range	18–60		18–60		18–59	
18–45	317	79.1	103	69.1	214	84.9
45–60	84	20.9	46	30.9	38	15.1
Sex						
Female	153	38.2	38	25.5	115	45.6
Male	248	61.8	111	74.5	137	54.4
cHL Subtype						
Mixed cellularity	116	28.9	65	43.6	51	20.2
Nodular sclerosis	257	64.1	74	49.7	183	72.6
Lymphocyte-rich	11	2.7	3	2	8	3.2
Lymphocyte-depleted	4	1	3	2	1	0.4
cHL NOS	13	3.2	4	2.7	9	3.6
IPS (grouped)						
0–1	114	28.4	29	19.5	85	33.7
2–3	224	55.9	92	61.7	132	52.4
4–7	63	15.7	28	18.8	35	13.9
Relapse/Death						
None	232	57.9	81	54.4	151	59.9
Progression/Relapse only	79	19.7	30	20.1	49	19.4
Death only	54	13.5	29	19.5	25	9.9
Both	36	9	9	6	27	10.7

B. Overall survival (OS)		
Variable	univar. P	multivar. P
Age	<0.0001	<0.0001
Sex (male)	0.0513	0.1759
adj. gene predictor	0.0079	0.525

C. Progression-free survival (PFS)		
Variable	univar. P	multivar. P
Age	0.0002	0.0006
Sex (male)	0.0014	0.0034
adj. gene predictor	0.1452	0.7881

D. OS and PFS with IPS		
Variable	OS	PFS
	multivar. P	multivar. P
IPS	0.0036	0.0027
adj. gene predictor	0.1438	0.7199

E. Adj. gene predictor and IPS risk factors		
Variable	stand. estimate β	significance P
Intercept	−0.98	<0.0001
Sex (male)	0.55	<0.0001
Age ≥ 45 yrs	0.46	<0.0001
Leukocytes ≥ 15,000/	0.66	<0.0001
Hb < 10.5 g/dL	0.52	<0.0001
Lymphocytes < 8% of WBC	0.39	0.0223
Albumin < 4 g/dL	−0.07	0.4928
Stage IV	−0.11	0.2328

Multiple linear regression of adjusted gene predictor on IPS factors, *R*^2^ = 0.22.

(**A**) Characteristics of high-risk and low-risk patients analyzed in this study. (**B**) Cox regression of progression-free survival (PFS) on sex, age and the adjusted gene predictor. (**C**) Cox regression of overall survival (OS) on sex, age and the adjusted gene predictor. (**D**) Cox regressions of PFS and OS on the IPS and the adjusted gene predictor. (**E**) Multiple linear regression of the adjusted gene predictor on the seven risk factors of the IPS.

We performed gene expression profiling of the underlying 404 samples using the NanoString nCounter platform. As a quality control measurement, we performed an inter-laboratory comparison of 24 randomly selected nCounter gene expression measurements from our cohort and further 24 samples from the initial study [2] (Supplementary Fig. 1). Samples were processed at two sites independently (Vancouver, Canada; Kiel, Germany) as described previously [2] (see also Supplementary methods). We observed an agreement between data generated at both institutions of *R*^2^ = 0.91 (Supplementary Fig. 2). The concordance of the published 23-gene outcome predictor scores between the two data sets was similar (*R*^2^ = 0.90, Supplementary Fig. 3, left panel). To further improve the transferability of the 23-gene predictor, we recalibrated its weights (see Supplementary methods). Here, we followed a previously published strategy^6^, and re-adjusted the signature weights such that the influence of data normalization on predictions is reduced (see also Supplementary methods, Supplementary Table 1, Supplementary Fig. 4). This step was necessary since the housekeeping genes in Vancouver and Kiel code sets differed and thus the respective normalization. For re-calibration, we used the same 23 genes of the published predictor and used gene expression levels of the original training data [2]. The adjusted gene predictor produced scores that correlated strongly with the original scores in the previously published Vancouver data set of both the training and the validation cohort (Supplementary Figs. 5, 6, respectively). The adjusted gene predictor compensated for the systematic differences between data generated in Vancouver and Kiel, yielding an excellent agreement between both data sets (*R*^2^ = 0.99, Supplementary Fig. 3, right panel).

**Fig. 1:**
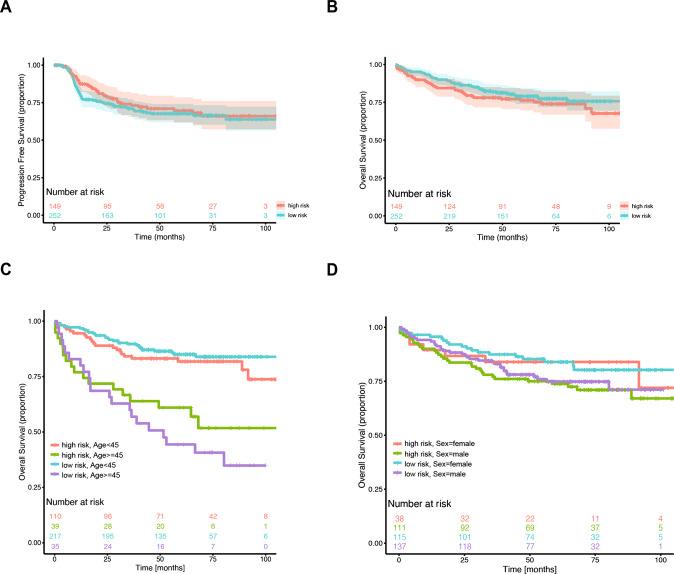
Kaplan–Meier survival curves for the 23-gene adjusted prognostic gene predictor in 401 patients with advanced stage cHL treated within clinical trials HD12 and HD15. **A** PFS comparing 23-gene adjusted prognostic gene predictor positive (red) and negative (blue) cohorts. **B** OS comparing 23-gene adjusted prognostic gene predictor positive (red) and negative (blue) cohorts. **C** OS comparing 23-gene adjusted prognostic gene predictor high-risk (red: age < 45; green: age ≥ 45) and low-risk (blue: age < 45; purple: age ≥ 45) cohorts. **D** OS comparing 23-gene adjusted prognostic gene predictor high-risk (red: female sex; green: male sex) and negative (blue: female sex; purple: male sex) cohorts.

Based on the predictions of the previously published 23-gene predictor [2], we separated our patient cohort into a low- and high-risk group (Table 1A). Notable differences occurred between both groups with respect to sex (male) and age (≥45 yrs), with male and older patients overrepresented in the high-risk group. The adjusted gene predictor did not differentiate high-risk cHL patients with respect to PFS (Fig. 1A, Cox model, *p* = 0.478), nor differentiated two groups of different OS (Fig. 1B, Cox model, *p* = 0.286) in BEACOPP-treated patients. In a multivariate analysis including age (≥45 yrs) and sex, neither OS nor PFS were significant (Cox model, OS: *p* = 0.299, PFS: *p* = 0.434). Higher age, but not male sex were significantly associated with worse OS; low- or high-risk scores did not further separate groups of patients with differential survival (Fig. 1C, D). Age was the most important prognostic variable for OS and PFS both in univariate and multivariate analysis, whereas sex was prognostic for PFS exclusively (Table 1B,C).

We next analyzed the role of the IPS, which consists of the risk factors sex (male), age (≥45 yrs), leukocytes ≥ 15,000/L, Hb < 10.5 g/dL, lymphocytes < 8% of WBC, stage IV and albumin < 4 g/dL, in our 401-patient cohort. We observed that the IPS was both significantly prognostic for OS and PFS in BEACOPP-treated patients (Table 1D, *p* = 0.0036 and *p* = 0.0027, respectively). The adjusted gene predictor was significantly associated with the following risk factors which are part of the IPS: sex, age, leukocytosis and anemia at diagnosis (Table 1E). Consequently, the adjusted gene predictor did not detect significant differences for PFS and OS when adjusted for IPS in our cHL cohort treated with BEACOPP-based regimens. Notably, the adjusted gene predictor was associated with death from acute toxicity (*p* = 0.022) and thus may rather describe tolerability of BEACOPP chemotherapy and the overall vulnerability of our patients.

We and others have recently shown that the cHL tumor microenvironment can be utilized as a prognostic tool in advanced stage cHL [5, 6]. Since in the context of BEACOPP treatment the published 23-gene predictor was not independently associated with outcome, we extended our analysis to an additional set of 119 genes. These genes were selected by involvement in Gene Ontology (GO) biological pathways associated with immune response and genes previously published to be associated with outcome in cHL (Supplementary Table 2, see reference list and selected GO terms in Supplementary Table 2). We tested for the association of gene-expression levels with OS and PFS by using Cox proportional hazard (PH) models on all available 401 samples. Cox PH models were trained by modelling each gene separately together with age, sex, and IPS discretized into IPS ≤ 2 and IPS > 2. Thus, gene-wise *p* values were adjusted for age, sex and IPS and corrected for by multiple testing. None of the 119 genes tested were associated with OS. Strikingly, however, *PDGFRA*, *TNFRSF8 (CD30)* and *CCL17 (TARC)* were significantly correlated with PFS (FDR < 0.05, Supplementary Table 3 for PFS, Supplementary Table 4 for OS). From a biological perspective, all three identified genes play a prominent role in cHL. TNFRSF8, also known as CD30, is typically expressed on Hodgkin Reed-Sternberg cells (HRSC) [7]. CD30 further serves as a target for the FDA-approved therapeutic brentuximab vedotin [7]. Moreover, platelet-derived growth factor receptor A (PDGFRA) is a receptor tyrosine kinase, and has also been shown to be expressed in HRSC and not in normal B cells, indicating a role for PDGFRA signaling in cHL pathogenesis [8]. Further, we and others have shown, that high baseline serum CCL17 (TARC) levels, are related to poor response in cHL [9, 10].

In order to build a prediction model for PFS, we separated our BEACOPP-treated cHL cohort into a training (HD15, 210 patients) and an independent validation cohort (HD12, 191 patients). The signature developed on the training cohort (Supplementary Table 5) consisted of CCL17 quantified relative to the four housekeeping genes. Next, we established a cutoff to separate high- from low-risk patients with corresponding KM curves shown in Supplementary Fig. 7 (Cox model, *p* < 0.001). These labels were also significantly associated with PFS in a multivariate CoxPH model together with age (≥45 yrs) and sex (Cox model, *p* < 0.001). We then applied our developed signature to the validation cohort, where we utilized the cutoff established on the training cohort to classify patients into high- and low-risk. Importantly, high- and low-risk patients significantly differed with respect to PFS (Supplementary Fig. 8, Cox model, *p* = 0.0059). This finding could be substantiated in a multivariate analysis together with age (≥45 yrs) and sex (Cox model, *p* = 0.0040). To increase statistical power, we combined HD12 and HD15 patients for further model development. The corresponding PFS model included *CCL17*, and additionally verified *PDGFRA*, and *TNFRSF8* for prognostication in our BEACOPP-treated cHL cohort. In line with our previous findings, this signature was prognostic for PFS (*p* < 0.001, Supplementary Table 6). We note that this model needs to be validated in independent cohorts in the future, while the former model was developed on a training cohort (HD15) and confirmed on an independent validation cohort (HD12).

In summary, the 23-gene predictor, which was previously shown to be a valuable prognostic tool in ABVD-treated patients, was not prognostic in a multivariate analysis for PFS and OS in the context of BEACOPP-treated advanced stage cHL. The most likely explanation is that the more dose-intense BEACOPP treatment itself overcomes the prognostic value of the gene predictor. In fact, the gene predictor seems to exert its potential only in the context of what it was originally designed for (ABVD-treated cHL) and transferring it to a different clinical context failed also in a previous attempt [11]. It is important to note, that the gene predictor was designed to predict OS, which is influenced by events not necessarily related to the aggressiveness of the lymphoma, such as death of other causes. Moreover, differences in PFS do not necessarily translate into differences in OS since effective therapy for relapsed disease is available [12]. Thus, from a clinical perspective, future biomarker development aiming at risk-stratification at diagnosis might also need to be aligned to PFS and interim PET/CT as an endpoint in cHL [13]. Moreover, different therapeutic approaches including Brentuximab-Vedotin and immune-checkpoint inhibition may generate the necessity to develop diverse predictors for risk assessment in cHL. The identification of *PDGFRA*, *TNFRSF8 (CD30)* and *CCL17 (TARC)* as single genes being prognostic for PFS after multiple testing in the BEACOPP-treated patient cohort highlights the potential of gene expression profiling for pre-treatment risk assessment. Despite the fact, that these three genes are likely mainly derived from HRSC, the abundance of HRSC by image analysis did not show a significant association with outcome in our previous study [5]. Since quantification of large cells, such as HRSC and macrophages, is tricky by quantitative image analysis [5], the relative cell content does not necessarily correlate with the expression level of genes derived from these cell types. This seems specifically true for secreted proteins as the ones identified in our analysis. Future studies are required to understand (i) how the number of HRSC and their potential to secrete immunologically active proteins such as *PDGFRA*, *TNFRSF8 (CD30)* and *CCL17 (TARC)* are associated with each other, (ii) which features mediate the aggressiveness of the disease under conventional chemotherapy and (iii) how these features can be assessed to identify patients suited for novel treatment strategies.

## Supplementary information

Supplementary material

Supplementary Figures 1-8

Supplementary Table 1

Supplementary Table 2

Supplementary Table 3

Supplementary Table 4

Supplementary Table 5

Supplementary Table 6
