# Supplementary material for: Gene expression-based outcome prediction in advanced stage classical Hodgkin lymphoma treated with BEACOPP
Source: Leukemia. 2021 Jun 10;35(12):3589–93. doi: 10.1038/s41375-021-01314-1 (PMC8632672; doi:10.1038/s41375-021-01314-1)
Supplement: Supplementary file 1 — Supplementary material [file 41375_2021_1314_MOESM1_ESM.docx]

**Supplementary Table and Figure Legends**

**Supplementary Figure 1.** Work flow of the intra-laboratory comparison, including pre-processing steps and subsequent batch effect analysis.

**Supplementary Figure 2.** Comparison of nCounter expression levels of the Vancouver batch (x-axis) and the Kiel batch (y-axis). Here, all samples are shown, including the two low quality samples shown as red. Expression levels correlate with R^2^= 0.91.

**Supplementary Figure 3.** The left figure shows the original gene predictor applied to the Vancouver batch (x-axis) and the Kiel batch (y-axis) (R^2^=0.90). The right figure shows the corresponding plot for the zero-sum gene predictor (R^2^=0.99).

**Supplementary Figure 4.** Work flow illustrating the adjustment of the 23-gene outcome predictor and its validation.

**Supplementary Figure 5.** Kaplan Meier curves for the training cohort of Scott *et al.*(1) stratified by the original 23-gene outcome predictor (left) and the adjusted 23-gene outcome predictor (right).

**Supplementary Figure 6.** Kaplan Meier curves for the validation cohort of Scott *et al.*(1) stratified by the original 23-gene outcome predictor (left) and the adjusted 23-gene outcome predictor (right).

**Supplementary Figure 7.** Kaplan Meier curves for the training cohort stratified by prognostic model of CCL17 quantified relative to the four housekeeping genes.

**Supplementary Figure 8.** Kaplan Meier curves for the validation cohort stratified by the identified prognostic model.

**Supplementary Table 1.** Comparison of adjusted signature vs. original signature.

**Supplementary Table 2.** Extended 119 gene immune response panel.

**Supplementary Table 3.** Extended 119 gene immune response panel correlation with progression-free survival (PFS) after correction for multiple testing.

**Supplementary Table 4.** Extended 119 gene immune response panel correlation with overall survival (OS) after correction for multiple testing.

**Supplementary Table 5.** Prognostic model developed on the HD15 cohort.

**Supplementary Table 6.** Prognostic model developed on the HD12 and HD15 cohort

**Supplementary Section/Methods**

**Pre-processing**

For the inter-laboratory comparison, we followed the protocol of Scott *et al.*(1) and corrected for hybridization variability across samples by multiplying the gene expression raw data by the mean sum of the positive spike-in controls across all the samples divided by the sum of the positive spike-in controls for that samples. Then we subtracted the mean of the negative controls. Finally, we divided the counts by the geometric mean of the reference genes, multiplied the result by 1000, added a pseudo count and transformed the data by taking the log2. For the Inter-laboratory comparison, we adjusted the global mean of the Kiel batch to that of the Vancouver batch to make the two batches more comparable quantitatively. In the BEACOPP cohort, three samples were removed due to low quality defined by total raw counts of endogenous and housekeeping genes less than 25000. The remaining pre-processing steps were done as above, except for the background correction, which was not required by data quality.

**The zero-sum outcome predictor**

Molecular biomarker signatures become robust with respect to batch-to-batch variability if their regression weights sum up to zero (2). We thus trained a zero-sum predictor that mimics the original predictor scores on the Vancouver training cohort (1). We performed the following steps:

(1) we learned a linear model which predicts the mean housekeeper expression levels (ACTB, CLTC and RPLP0), using the expression levels of the signature genes as predictor variables:

Let $x_{ij}$ be the log-expression level of gene $i$ in sample $j$ and $\bar{x}_{HK,j}=\frac{1}{q}\sum_{i\in HK} x_{ij}$ the mean housekeeper (HK) expression level, where $q$ is the number of HK genes. We minimized $\sum_{j} (\bar{x}_{HK,j}-\beta_{0}-\sum_{i\epsilon A} \beta_{i}{x_{ij})}^{2}$ while keeping the sum of the regression weights equal to 1, i.e. $\sum_{i\epsilon A} \beta_{i}=1$. This yields a linear model to replace the housekeeper normalization. Note that normalization to the geometric mean corresponds to a subtraction of the mean HK expression on log scale. The constraint $\sum_{i\epsilon A} \beta_{i}=1$ was incorporated to ensure invariance under rescaling the profiles, i.e. $x_{ij}\to x_{ij}+ \gamma_{j}$.

(2) we used the molecular signature trained in (1) to turn the 23-gene outcome predictor into a “zero-sum” form:

The predictor score of the original 23-gene signature is of the form ${y_{j}=\beta}_{0}+\sum_{i\epsilon A} \beta_{i}\tilde{x}_{ij}$, where $\tilde{x}_{ij}=x_{ij}-$ $\bar{x}_{HK,j}$. We replaced $\bar{x}_{HK,j}$ by the linear model of (1), which yields a molecular signature $\tilde{\beta}_{0}+\sum_{i\epsilon A} \tilde{\beta}_{i}x_{ij}\approx\beta_{0}+\sum_{i\epsilon A} \beta_{i}\tilde{x}_{ij}=y_{j}$, where $\sum_{i\epsilon A} \tilde{\beta}_{i}=0$. Note that zero-sum signatures do not require any sample-wise scaling of the data. The predictions become independent of the applied scaling protocol and signature weights do not need to be readjusted if the signature is applied to data from another technology (2). The latter requires only the adjustment of the model’s intercept $\beta_{0}$.

**Development of a prognostic model for BEACOPP-treated cHL patients**

We performed Cox lasso regression on PFS using endogenous genes as predictor variables, where the data were preprocessed as outlined above. We performed a cross-validation for different values of the regularization parameter $\lambda$. We retrieved the model with parameter choice “lambda.1se”, provided in the R-package glmnet (3), where we optimized the concordance index (C-Index). The model developed on the HD15-BEACOPP cohort is summarized in Supplementary Table 5. The cutoff to separate low- from high-risk patients was established on HD15 based on the lowest p-value in a CoxPH model. The joint model based on HD12 and HD15 is summarized in Supplementary Table 6. Here, low- and high-risk patients were obtained by cutting the score at the median. Note, both developed signatures have the aforementioned zero-sum property. The corresponding p-values were extracted using Cox PH models employing the risk categories.

**References**

1. Scott DW, Chan FC, Hong F, Rogic S, Tan KL, Meissner B, et al. Gene expression-based model using formalin-fixed paraffin-embedded biopsies predicts overall survival in advanced-stage classical Hodgkin lymphoma. J Clin Oncol. 2013;31(6):692-700.

2. Altenbuchinger M, Schwarzfischer P, Rehberg T, Reinders J, Kohler CW, Gronwald W, et al. Molecular signatures that can be transferred across different omics platforms. Bioinformatics. 2017;33(14):i333-i40.

3. Simon N, Friedman J, Hastie T, Tibshirani R. Regularization Paths for Cox's Proportional Hazards Model via Coordinate Descent. J Stat Softw. 2011;39(5):1-13.
