# Supplementary material for: Gene expression-based outcome prediction in advanced stage classical Hodgkin lymphoma treated with BEACOPP
Source: Leukemia. 2021 Jun 10;35(12):3589–93. doi: 10.1038/s41375-021-01314-1 (PMC8632672; doi:10.1038/s41375-021-01314-1)

Supplementary Figure 1

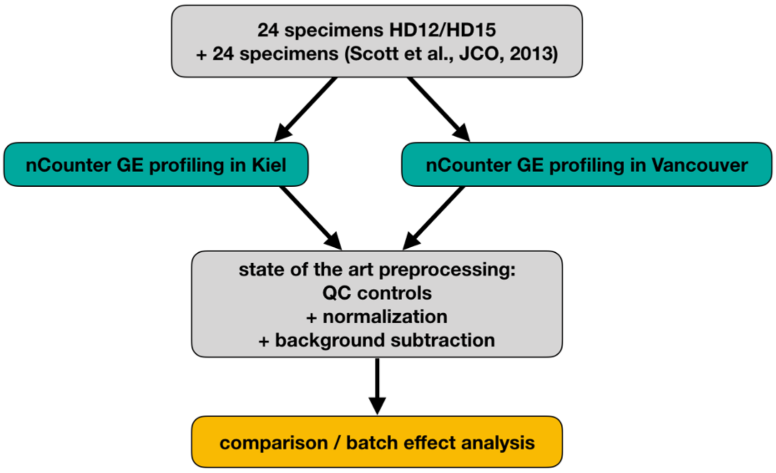

Supplementary Figure 2

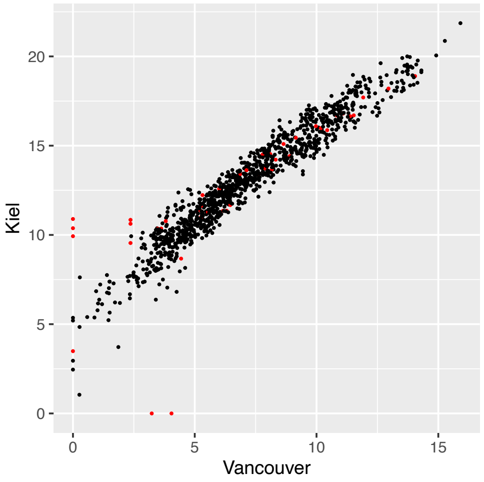

Supplementary Figure 3

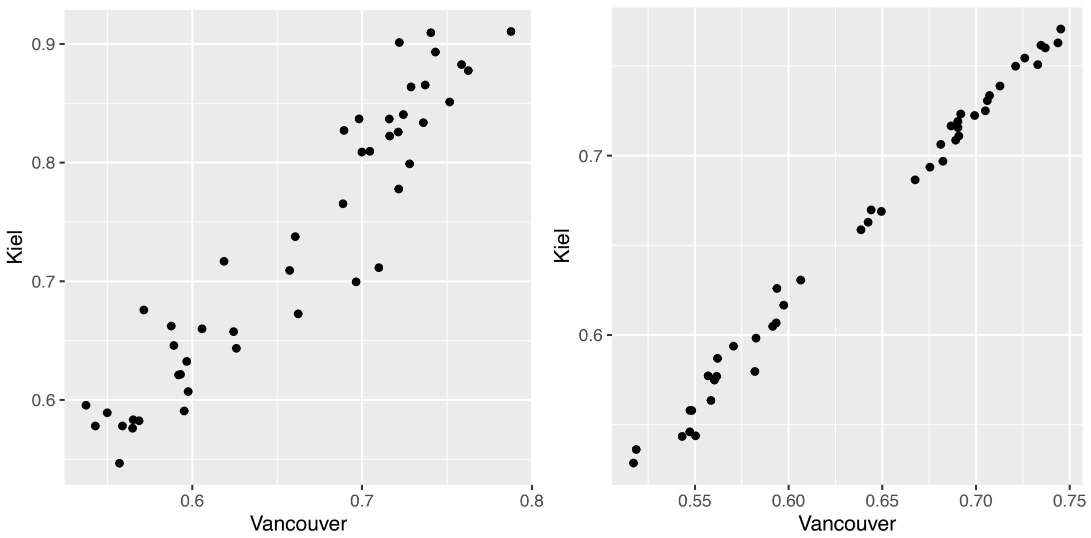

Supplementary Figure 4

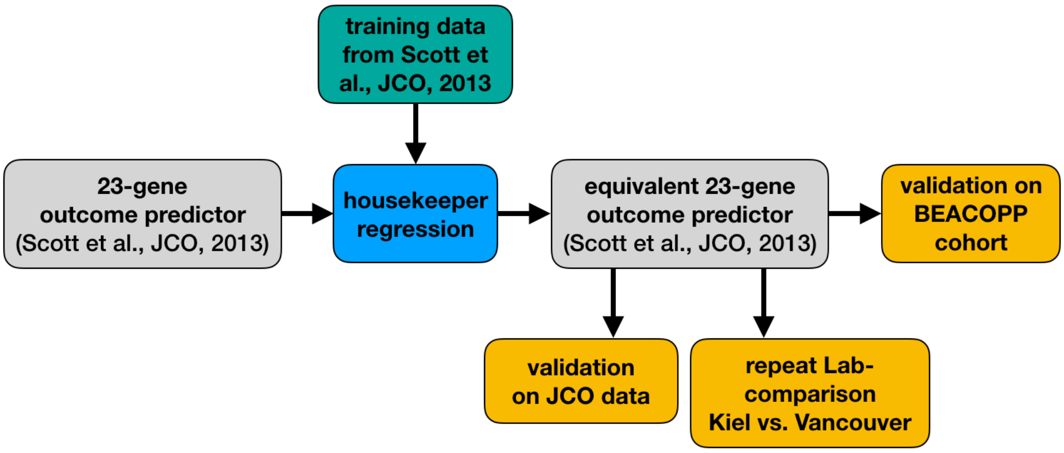

Supplementary Figure 5

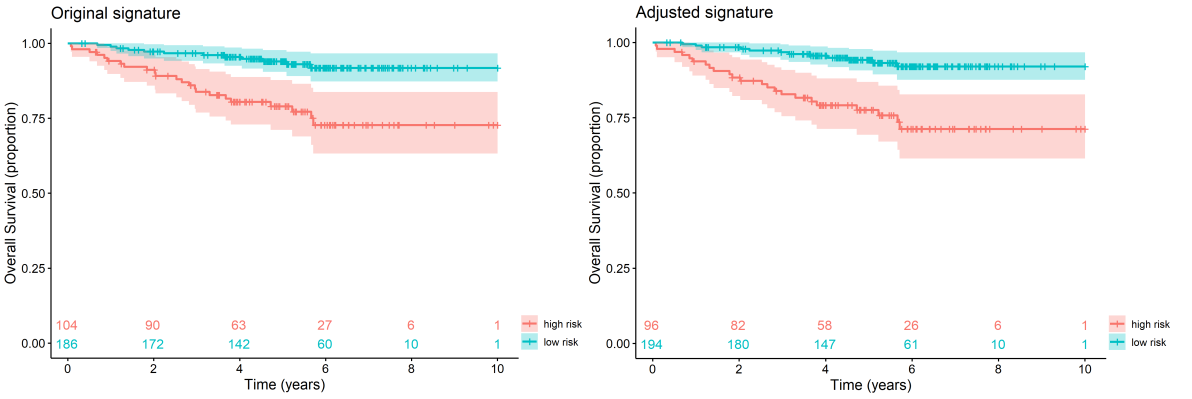

Supplementary Figure 6

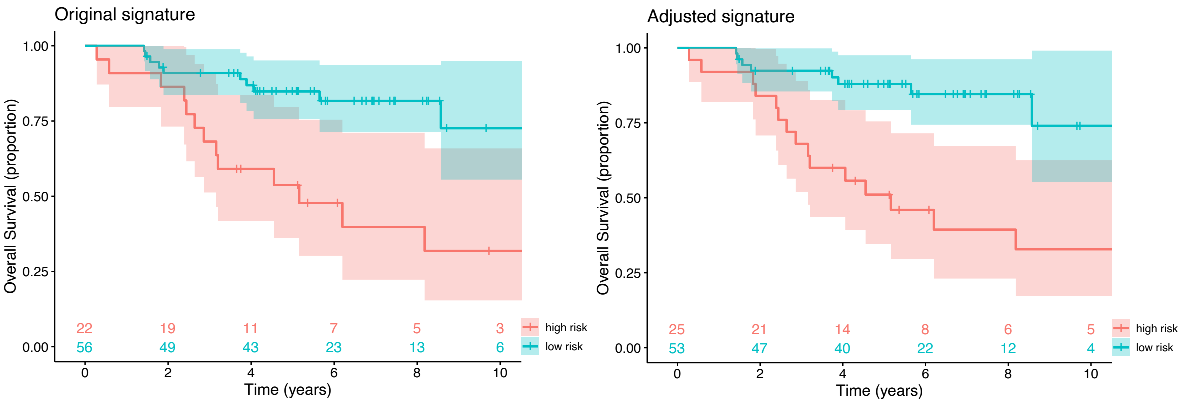

Supplementary Figure 7

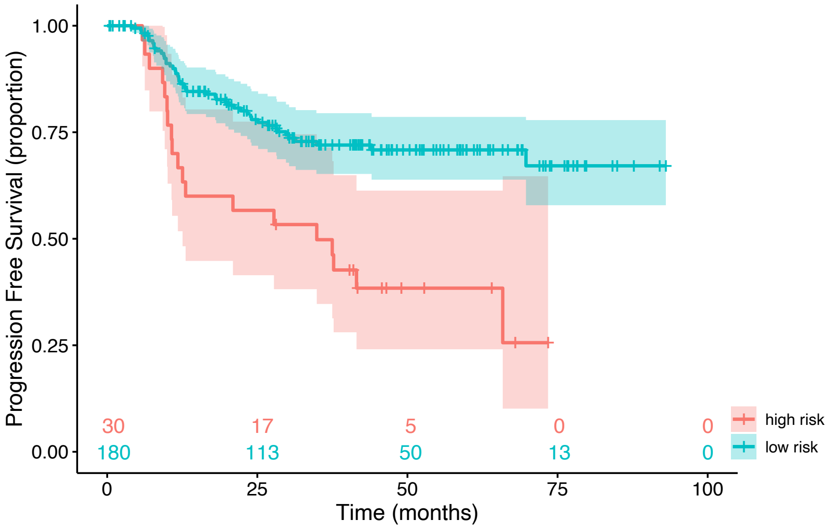

Supplementary Figure 8

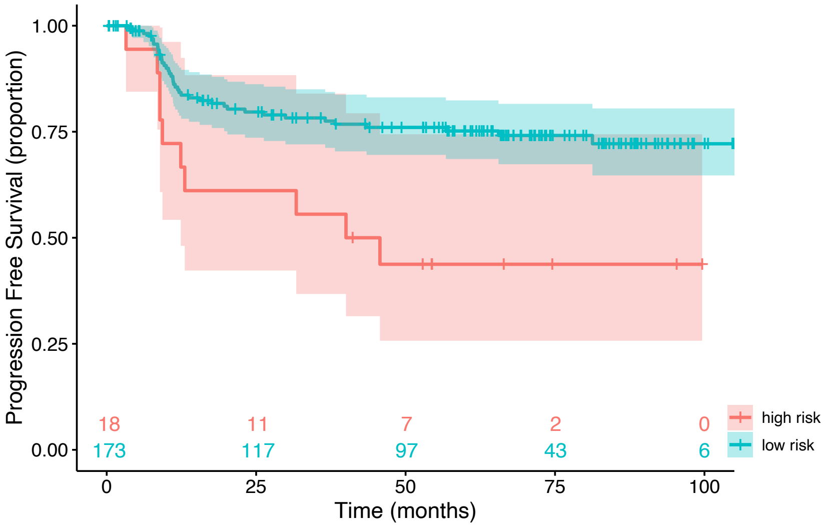

Supplement: Supplementary file 2 — Supplementary Figures 1-8 [file 41375_2021_1314_MOESM2_ESM.pdf]
